# Association between psoriasis and colorectal cancer: A meta-analysis

**DOI:** 10.7555/JBR.39.20250175

**Published:** 2025-07-25

**Authors:** Yufei Wang, Jiliang Lu, Ziyue Diao, Zhiqiang Yin

**Affiliations:** Department of Dermatology, The First Affiliated Hospital of Nanjing Medical University, Nanjing, Jiangsu 210029, China

Dear Editor,

Psoriasis is increasingly recognized as a systemic inflammatory disease associated with several comorbidities, including metabolic syndrome, depression, and malignancies^[1]^. Colorectal cancer (CRC) is the third most common cancer worldwide and ranks second in mortality among all malignancies. Currently, it has become one of the most severe challenges faced by healthcare systems in many countries^[2]^. A previous study has found that patients with psoriasis have a significantly increased risk of developing CRC^[3]^; however, the stratified characteristics between them are unclear. We performed a meta-analysis of observational studies examining the relationship between psoriasis and CRC. An Embase and Medline search was conducted on January 19, 2025, which identified 377 relevant studies. The search strategy and PRISMA study flow chart are presented in ***Supplementary Table 1*** and ***Supplementary Fig. 1***. Following screening, a total of 10 relevant articles were selected for inclusion. We extracted study design, first author's last name, publication year, population information, and risk estimates (*i.e.*, hazard ratio [HR], incidence rate ratio [IRR], risk ratio [RR], and odds ratio [OR]) with 95% confidence intervals (CIs) for the association between psoriasis and CRC (***Supplementary Table 2***). The Newcastle-Ottawa Scale was used to assess the risk of bias in the included studies (***Supplementary Fig. 2***). Data analysis was conducted using RevMan 5.4. The inverse-variance weighting method, random-effects model, and *I*^2^ statistics for heterogeneity were used for analysis (***Supplementary Fig. 3***).

The results indicated a significantly increased risk of CRC among patients with psoriasis (HR = 1.22, 95% CI: 1.11–1.34), with no significant heterogeneity observed across studies. Sensitivity analyses confirmed the robustness of our primary findings (***Supplementary Fig. 4***). Subgroup analyses revealed a higher risk in American psoriatic patients (HR = 1.49, 95% CI: 1.08–2.04), followed by Asian patients (HR = 1.41, 95% CI: 1.09–1.83), and a moderate increase in European patients (HR = 1.14, 95% CI: 1.06–1.22) (***Fig. 1***). In the subgroup analysis by sex (***Fig. 2***), no significant association was found between psoriasis and CRC among male patients (HR = 1.19, 95% CI: 0.94–1.50), whereas an elevated risk was observed in female patients (HR = 1.51, 95% CI: 1.24–1.84), which is consistent with the findings of a previous study^[3]^. In the subgroup analysis by severity, neither mild psoriasis (HR = 1.16, 95% CI: 0.88–1.52) nor moderate-to-severe psoriasis (HR = 1.29, 95% CI: 0.91–1.81) was significantly associated with CRC (***Fig. 3***).

**Figure 1:**
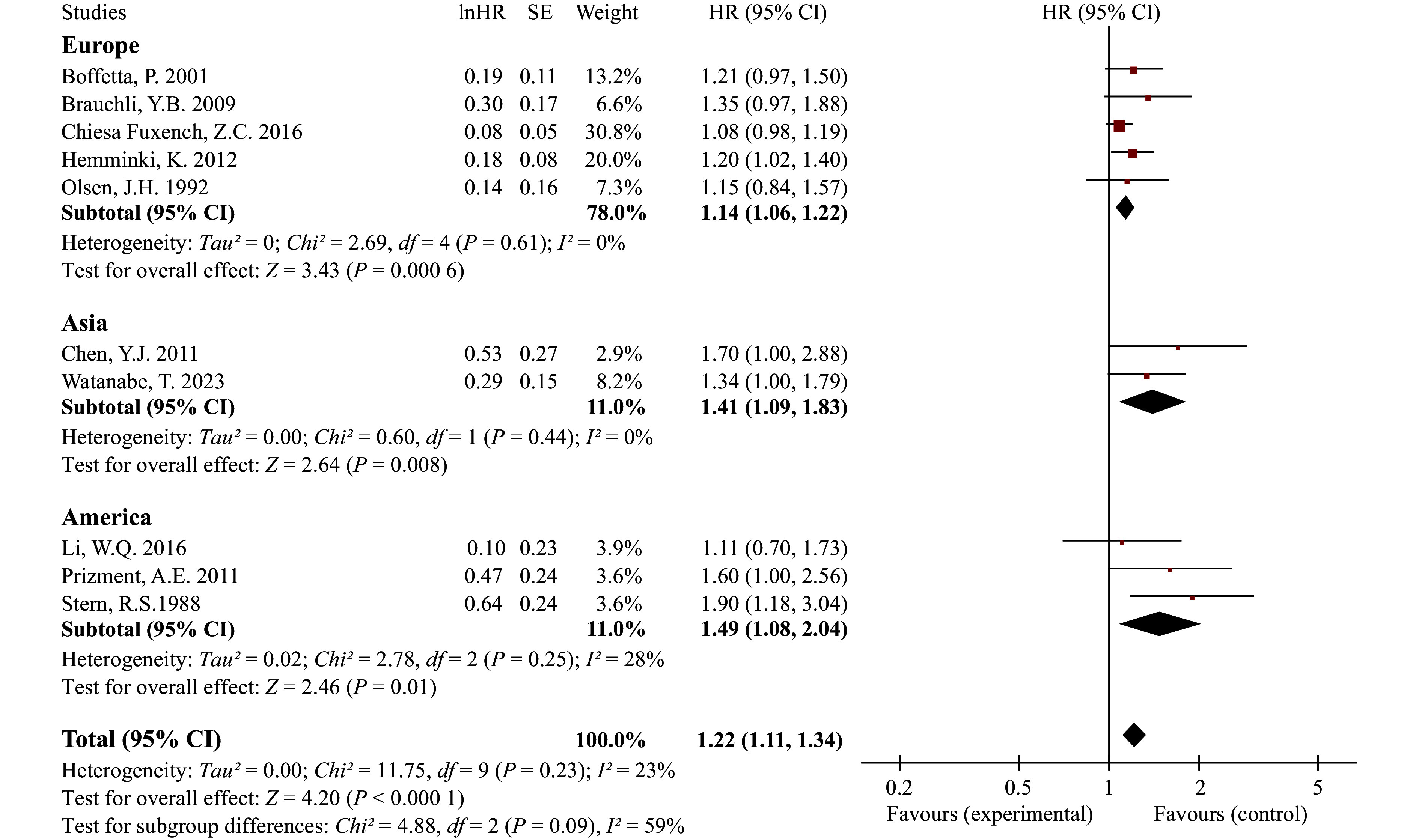
Forest plot for cohort studies on the association between psoriasis and colorectal cancer stratified by region. Statistical method: inverse-variance weighting method, random-effects model justification. Abbreviations: CI, confidence interval; df: degrees of freedom; HR, hazard ratio; SE, standard error.

**Figure 2:**
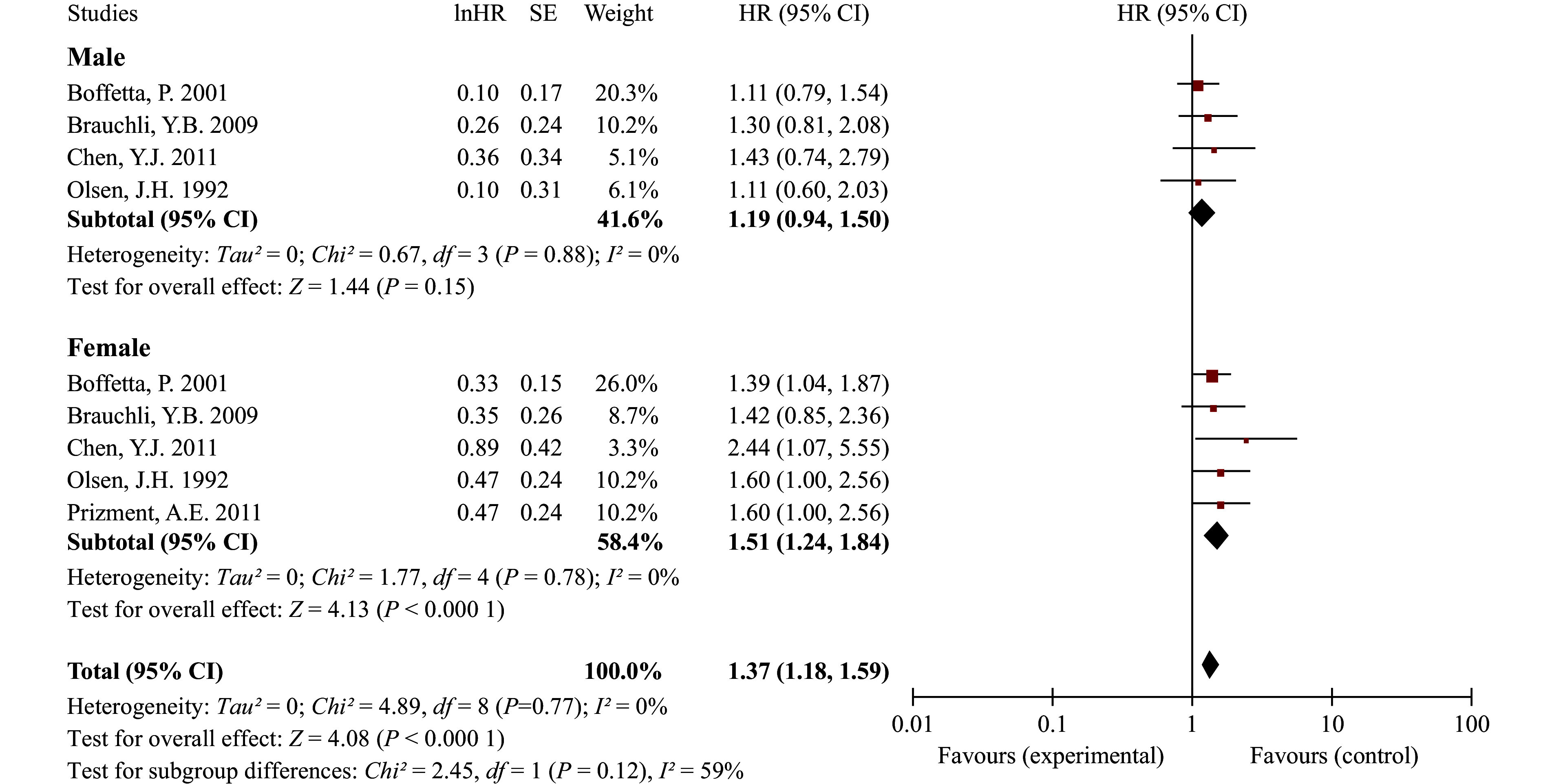
Forest plot for cohort studies on the association between psoriasis and colorectal cancer stratified by sex. Statistical method: inverse-variance weighting method, random-effects model justification. Abbreviations: CI, confidence interval; df: degrees of freedom; HR, hazard ratio; SE, standard error.

**Figure 3:**
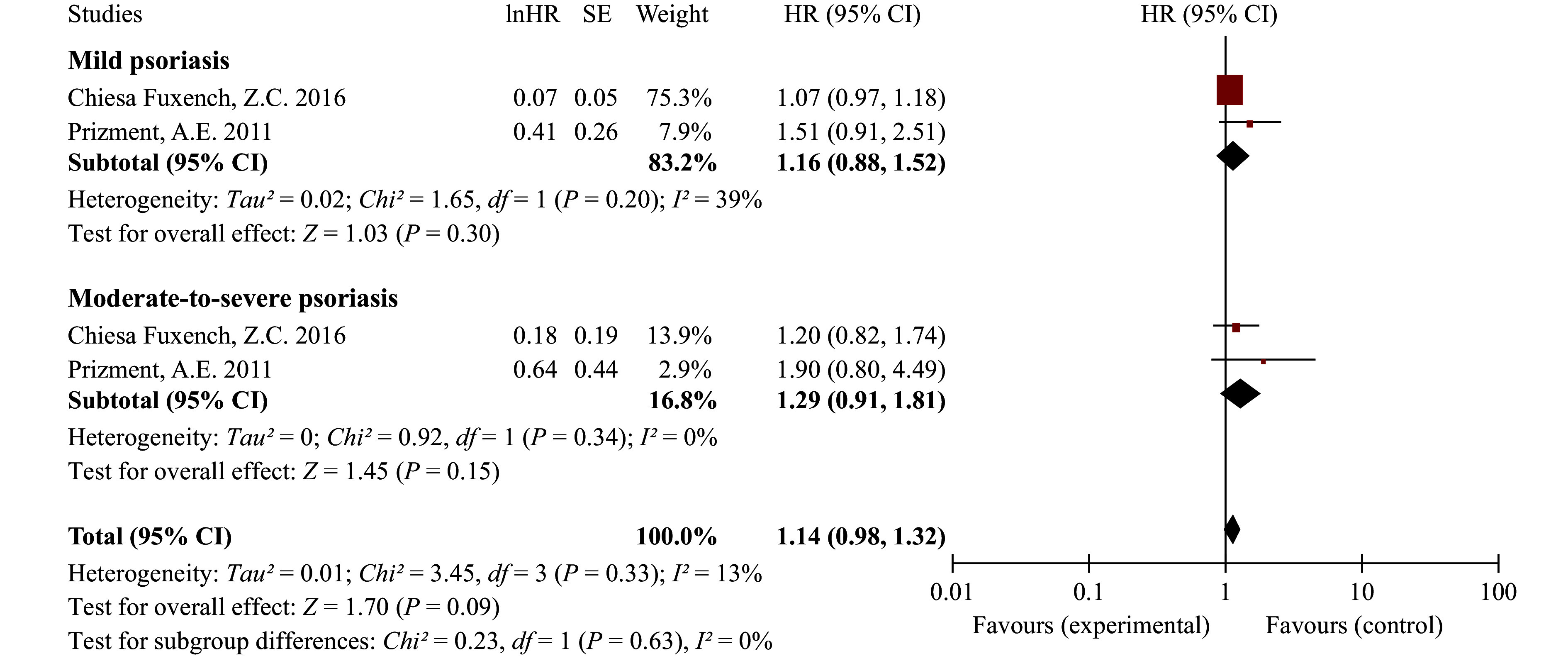
Forest plot for cohort studies on the association between psoriasis and colorectal cancer stratified by severity. Statistical method: inverse-variance weighting method, random-effects model justification. Abbreviations: CI, confidence interval; df: degrees of freedom; HR, hazard ratio; SE, standard error.

A recent study by GLOBOCAN on the incidence of CRC found that the highest rates were observed in Europe and Australia/New Zealand in 2020, with the global burden of CRC projected to increase to 3.2 million new cases by 2040^[4]^. Our study found that psoriatic patients in the Americas and Asia faced higher risks of developing CRC, compared with those in European regions. One previous study identified significant sex disparities in CRC incidence, showing an overall higher incidence among male populations, with males aged 50–59 and 60–69 years exhibiting particularly elevated disease risk^[5]^. Our study revealed that female psoriasis patients had a significantly higher risk of CRC, but this association was not observed in males. In summary, the incidence of CRC among psoriatic patients varies by region and sex, differing from that of the general population. This suggests that the development of CRC in individuals with psoriasis involves unique and complex mechanisms. A study revealed that postmenopausal women undergoing hormone therapy exhibited an increased risk of psoriasis, hinting at a potential link between estrogen and psoriasis pathogenesis^[6]^. Elevated estrogen levels in females may exacerbate psoriatic inflammation, potentially fostering colorectal tumorigenesis. It has been demonstrated that IL-17 plays a dominant role in the pathogenesis of psoriasis; moreover, IL-17 from various cellular sources promotes the development and progression of CRC^[7]^. Additionally, psoriasis treatment protocols, including the systemic use of multiple immunosuppressants, biologics such as TNF inhibitors, and small-molecule drugs, might influence CRC risk, and one study recommends a colonoscopy before initiating biological therapy^[8]^. Psoriatic patients from different regions exhibit distinct genetic backgrounds, and there is heterogeneity within their neuro-immuno-endocrine networks. The presence of psoriasis-associated comorbidities, such as inflammatory bowel disease, along with dietary variations, particularly in alcohol consumption, may collectively influence CRC development. A study based on the Global Burden of Disease database revealed substantial heterogeneity in the disease burden of psoriasis across countries, with the inequality related to the level of socio-demographic development worsening over time^[9]^. The high-fat dietary patterns prevalent in the Americas may exacerbate systemic chronic inflammation, which, combined with genetic predisposition^[10]^, could collectively influence cancer incidence rates. The underlying mechanisms and the influence of interpopulation genetic variations require further investigation to fully elucidate these associations.

In conclusion, this study confirms an increased incidence of CRC among psoriatic patients and observes significant regional variations in this risk across global populations, with a particularly notable association identified in female patients. Several limitations should be acknowledged, including the lack of CRC severity-stratification data, partial inclusion of outdated datasets, and the absence of treatment regimen documentation in psoriatic cohorts. These constraints underscore the necessity for future prospective cohort studies.

This work was supported by the National Natural Science Foundation of China (Grant No. 82373475).

Yours sincerely,

Yufei Wang^△^, Jiliang Lu^△^, Ziyue Diao^△^, 

Zhiqiang Yin^✉^

Department of Dermatology, the First Affiliated Hospital of Nanjing Medical University, 

Nanjing, Jiangsu 210029,

China.

^△^These authors contributed equally to this work.

^✉^Corresponding author: Zhiqiang Yin. E-mail: yinzhiqiang@njmu.edu.cn.

## Additional information

The online version contains supplementary materials available at http://www.jbr-pub.org.cn/article/doi/10.7555/JBR.39.20250175?pageType=en.
