# Supplementary material for: Association between psoriasis and colorectal cancer: A meta-analysis
Source: J Biomed Res. 2025 Jul 25;40(1):93–6. doi: 10.7555/JBR.39.20250175 (PMC12794175; doi:10.7555/JBR.39.20250175)
Supplement: Supplementary file 1 — The online version contains supplementary materials available at http://www.jbr-pub.org.cn/article/doi/10.7555/JBR.39.20250175?pageType=en. [file jbr-40-1-93-S1.pdf]

Available online at www.jbr-pub.org.cn

Open Access at PubMed Central

Journal of Biomedical Research, 2026 40(1): 1–4

Supplementary Data

JBR

Association between psoriasis and colorectal cancer: a meta-analysis

Yufei Wang, Jiliang Lu, Ziyue Diao, Zhiqiang Yin✉

Department of Dermatology, the First Affiliated Hospital of Nanjing Medical University, Nanjing, Jiangsu 210029, China.

Supplementary Table 1 **The search strategy of database**

Database		Query	Results
Embase	#5	#4 AND #1	169
Embase	#4	#2 OR #3	310 034
Embase	#3	'colorectal tumor':ti,ab,kw OR 'colorectal neoplasm':ti,ab,kw OR 'colorectal cancer':ti,ab,kw OR 'colorectal carcinoma':ti,ab,kw	236 504
Embase	#2	'colon tumor':ti,ab,kw OR 'colon neoplasms':ti,ab,kw OR 'colon cancer':ti,ab,kw OR 'colonic cancer':ti,ab,kw OR 'colon adenocarcinoma':ti,ab,kw	103 192
Embase	#1	'psoriasis':ti,ab,kw OR 'pustulosis palmaris et plantaris':ti,ab,kw OR 'psoriasis vulgaris':ti,ab,kw OR 'palmoplantaris pustulosis':ti,ab,kw OR ('pustular psoriasis of palms':ti,ab,kw AND soles:ti,ab,kw) OR 'psoriatic epidermis':ti,ab,kw	86 730
Pubmed	5	(#3) AND (#4)	202
Pubmed	4	(#1) OR (#2)	376 265
Pubmed	3	(Psoriasis) OR (Psoriases) OR (Pustulosis, Palms AND Soles) OR (Pustulosis Palmaris et Plantaris) OR (Palmoplantaris Pustulosis) OR (Pustular Psoriasis, Palms AND Soles)	69 225
Pubmed	2	((((((((((((((((((Colonic Neoplasm) OR (Neoplasm, Colonic)) OR (Colon Neoplasms)) OR (Colon Neoplasm)) OR (Neoplasm, Colon)) OR (Neoplasms, Colon)) OR (Neoplasms, Colonic)) OR (Cancer of Colon)) OR (Colon Cancers)) OR (Cancer of the Colon)) OR (Colonic Cancer)) OR (Cancer, Colonic)) OR (Cancers, Colonic)) OR (Colonic Cancers)) OR (Colon Cancer)) OR (Cancer, Colon)) OR (Cancers, Colon)) OR (Colon Adenocarcinoma)) OR (Adenocarcinoma, Colon)) OR (Colon Adenocarcinomas)) OR (Adenocarcinomas, Colon)	186 061
Pubmed	1	(((((((((((((Colorectal Neoplasm) OR (Neoplasm, Colorectal)) OR (Colorectal Tumors)) OR (Colorectal Tumor)) OR (Tumor, Colorectal)) OR (Tumors, Colorectal)) OR (Neoplasms, Colorectal)) OR (Colorectal Cancer)) OR (Cancer, Colorectal)) OR (Cancers, Colorectal)) OR (Colorectal Cancers)) OR (Colorectal Carcinoma)) OR (Carcinoma, Colorectal)) OR (Carcinomas, Colorectal)) OR (Colorectal Carcinomas)	324 110

✉Corresponding author: Zhiqiang Yin. E-mail: yinzhiqiang@njmu.edu.cn.

Received: 20 April 2025; Revised: 08 July 2025; Accepted: 11 July 2025; Published online: 25 July 2025

CLC number: R758.63, Document code: B

The authors reported no conflict of interests.

This is an open access article under the Creative Commons Attribution (CC BY 4.0) license, which permits others to distribute, remix, adapt and build upon this work, for commercial use, provided the original work is properly cited.

© 2026 by Journal of Biomedical Research.

https://doi.org/10.7555/JBR.39.20250175

Supplementary Table 2 **Information of the included studies**

Authors	Years	Study design	Country and population information	Risk (95% CI) for colorectal cancer	Reference
Boffetta, P.	2001	Cohort study	Sweden; 93 776 person-years	1.21 (0.97, 1.51)	[1]
Brauchli, Y. B.	2009	Cohort study	UK; 36 702 with psoriasis and 36 702 matched psoriasis-free patients	IRR = 1.35 (0.97, 1.90)	[2]
Chen, Y. J.	2011	Cohort study	Taiwan; 3 686 patients with psoriasis between 1996 and 2000. Another 200 000 patients without psoriasis served as the comparison group.	1.7 (1.01, 2.86)	[3]
Chiesa Fuxench, Z. C.	2016	Cohort study	UK; 198 366 patients with psoriasis and 937 716 without psoriasis	1.08 (0.98, 1.20)	[4]
Hemminki, K.	2012	Cohort study	Sweden; 15 592 patients with psoriasis	1.20 (1.03, 1.40)	[5]
Olsen, J.H.	1992	Cohort study	Denmark; 6 910 patients with psoriasis	RR = 1.15 (0.84, 1.57)	[6]
Li, W.Q.	2016	Cohort study	US; 1 404 patients with psoriasis and 63 586 without psoriasis	1.11 (0.71, 1.74)	[7]
Prizment, A. E.	2011	Cohort study	US; 719 patients with psoriasis and 32 191 without psoriasis	HR=1.6 (1.0, 2.4)	[8]
Stern, R. S.	1988	Cohort study	US; 1 380 patients with psoriasis	1.90 (1.18, 3.06)	[9]
Watanabe, T.	2023	Cohort study	Japan; 2 021 360 patients with psoriasis	2.55 (1.53, 4.25)	[10]

Abbreviations: CI, confidence interval; HR, hazard ratio; IRR, incidence rate ratio; OR, odds ratio; RR, risk ratio.

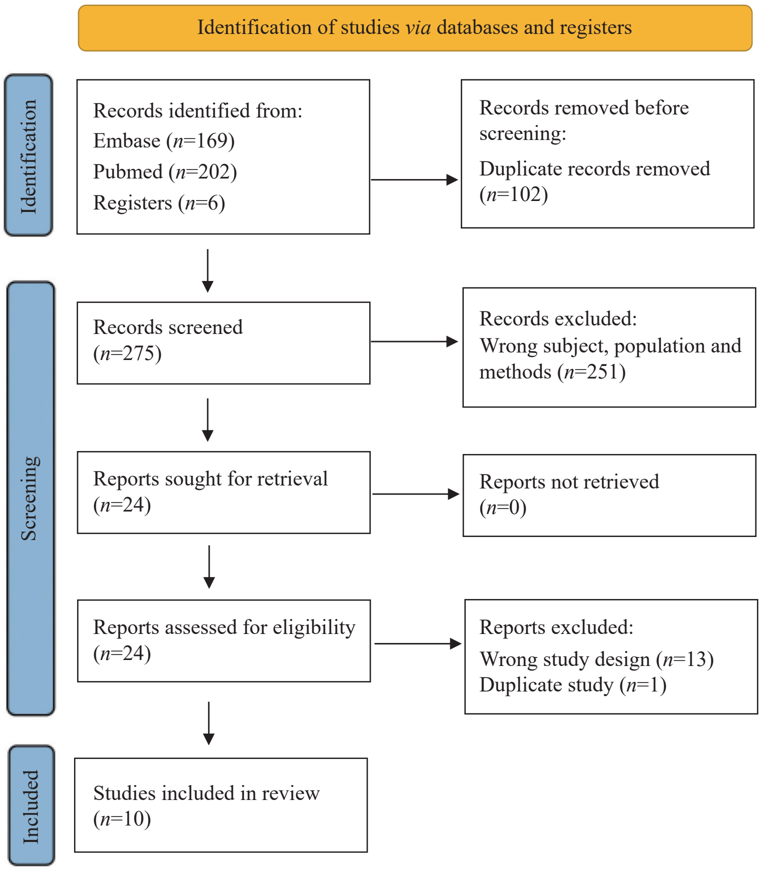

Supplementary Fig. 1 **The PRISMA study flow chart.** The PRISMA 2020 flow diagram for new systematic reviews, which included searches of databases and registers only.

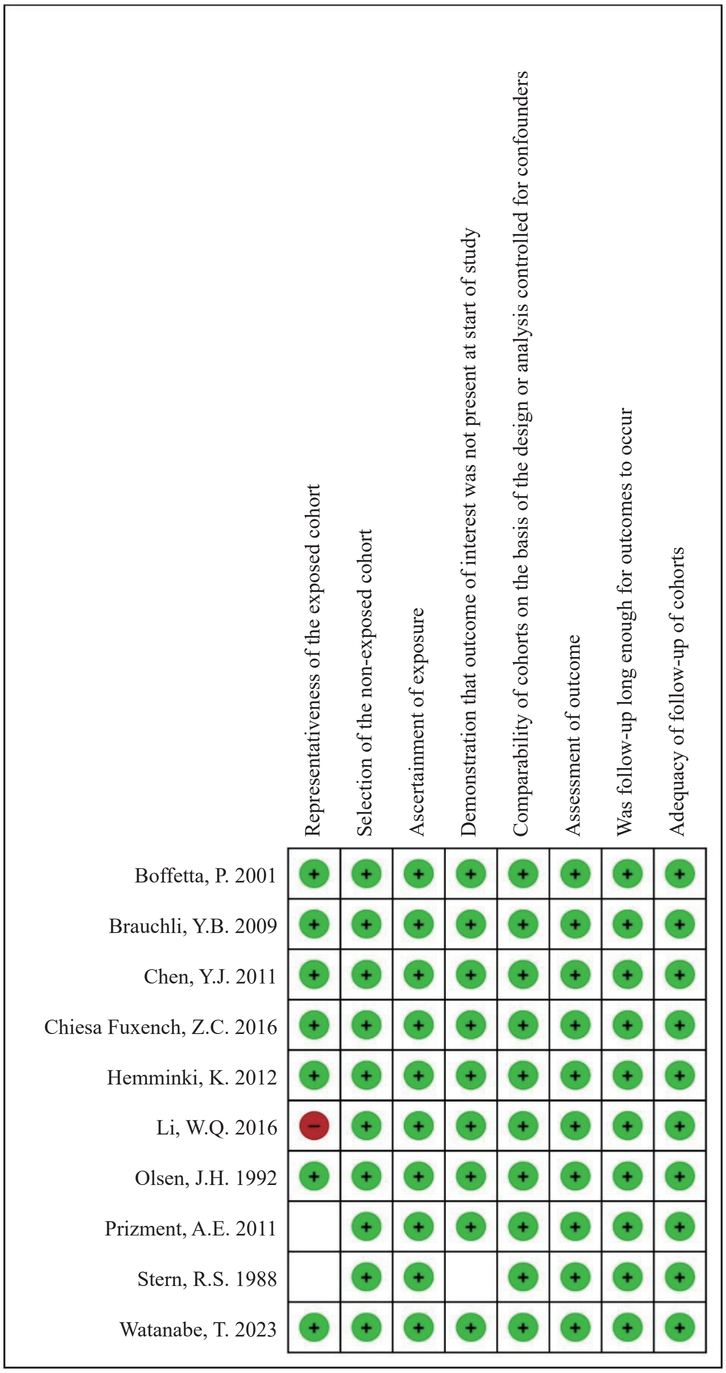

Supplementary Fig. 2 **Risk of bias summary of the included studies based on the Newcastle-Ottawa Scale (NOS).** + indicates high risk of bias; — indicates low risk of bias.

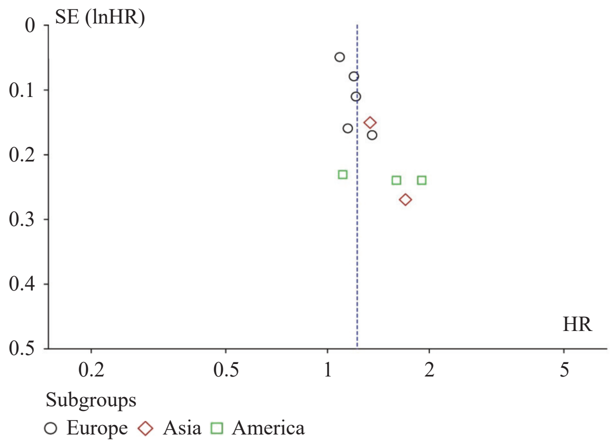

Supplementary Fig. 3 **Funnel plot of the included studies.** Abbreviations: HR, hazard ratio; SE, standard error.

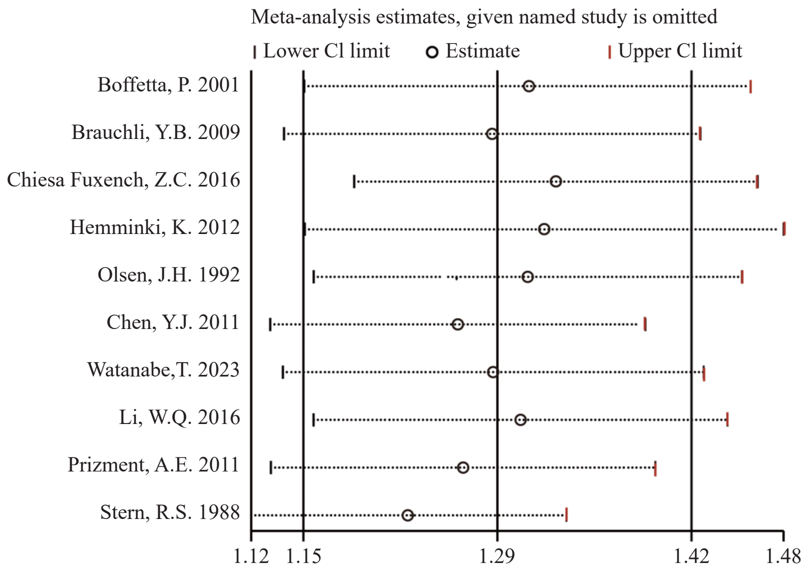

Supplementary Fig. 4 **Sensitivity analysis of the included studies.** Abbreviations: HR, hazard ratio; CI, confidence interval.

References

[1] Boffetta P, Gridley G, Lindelöf B. Cancer risk in a population-based cohort of patients hospitalized for psoriasis in Sweden[J]. *J Invest Dermatol*, 2001, 117(6): 1531–1537.

[2] Brauchli YB, Jick SS, Miret M, et al. Psoriasis and risk of incident cancer: An inception cohort study with a nested case-control analysis[J]. *J Invest Dermatol*, 2009, 129(11): 2604–2612.

[3] Chen Y, Wu C, Chen TJ, et al. The risk of cancer in patients with psoriasis: A population-based cohort study in Taiwan[J]. *J Am Acad Dermatol*, 2011, 65(1): 84–91.

[4] Chiesa Fuxench ZC, Shin DB, Ogdie Beatty A, et al. The risk of cancer in patients with psoriasis: A population-based cohort study in the health improvement network[J]. *JAMA Dermatol*, 2016, 152(3): 282–290.

[5] Hemminki K, Liu X, Ji J, et al. Autoimmune disease and subsequent digestive tract cancer by histology[J]. *Ann Oncol*, 2012, 23(4): 927–933.

[6] Olsen JH, Møller H, Frentz G. Malignant tumors in patients with psoriasis[J]. *J Am Acad Dermatol*, 1992, 27(5): 716–722.

[7] Li W, Han J, Cho E, et al. Personal history of psoriasis and risk of incident cancer among women: A population-based cohort study[J]. *Br J Dermatol*, 2016, 174(5): 1108–1111.

[8] Prizment AE, Alonso A, Folsom AR, et al. Association between psoriasis and incident cancer: The Iowa's Women's Health Study[J]. *Cancer Causes Control*, 2011, 22(7): 1003–1010.

[9] Stern RS, Lange R. Cardiovascular disease, cancer, and cause of death in patients with psoriasis: 10 years prospective experience in a cohort of 1380 patients[J]. *J Invest Dermatol*, 1988, 91(3): 197–201.

[10] Watanabe T, Watanabe Y, Asai C, et al. Risks of malignancies among patients with psoriasis: A cohort study of 360 patients[J]. *J Dermatol*, 2023, 50(5): 615–621.
